# Aqueous semisynthesis of *C*-glycoside glycamines from agarose

**DOI:** 10.3762/bjoc.13.121

**Published:** 2017-06-23

**Authors:** Juliana C Cunico Dallagnol, Alexandre Orsato, Diogo R B Ducatti, Miguel D Noseda, Maria Eugênia R Duarte, Alan G Gonçalves

**Affiliations:** 1 Departamento de Farmácia, Universidade Federal do Paraná, Avenida Lothario Meissner, 3400, Curitiba, Paraná, Brazil; 2 Departamento de Química, Universidade Estadual de Londrina, Rodovia Celso Garcia Cid (Pr 445), Km 380, PO Box 10011, Londrina, Paraná, Brazil; 3 Departamento de Bioquímica e Biologia Molecular, Universidade Federal do Paraná, Avenida Francisco H. dos Santos, 120, PO Box 19046, Curitiba, Paraná, Brazil

**Keywords:** amino sugar, 3,6-anhydro-α*-*L-galactopyranose, polysaccharide (agar), protecting-group-free, reductive amination

## Abstract

Agarose was herein employed as starting material to produce primary, secondary and tertiary *C*-glycoside glycamines, including mono- and disaccharide structures. The semisynthetic approach utilized was generally based on polysaccharide-controlled hydrolysis followed by reductive amination. All reactions were conducted in aqueous media and without the need of hydroxyl group protection. We were able to identify optimal conditions for the reductive amination of agar hydrolysis products and to overcome the major difficulties related to this kind of reaction, also extending it to reducing anhydrosugars. The excess of ammonium acetate, methyl- or dimethylamine, and the use of a diluted basic (pH 11) reaction media were identified as important aspects to achieve improved yields, as well as to decrease the amount of byproducts commonly related to reductive amination of carbohydrates. This strategy allowed the transposition of the 3,6-anhydro-α-L-galactopyranose unit (naturally present in the agarose structure) to all glycamines synthesized, constituting an amino-substituted *C*-threofuranoside moiety, which is closely related to (+)-muscarine.

## Findings

Agarose (**1**) is the term utilized to describe a type of galactose-containing polysaccharides biosynthesized by certain species of algae belonging to the division *Rhodophyta* (red seaweeds). It is a linear biopolymer constituted of the disaccharide repeating unit (1→3)-β-D-galactopyranose-(1→4)-3,6-anhydro-α-L-galactopyranose [1]. Both **1** and its less refined versions (agar or agar-agar) are well known due to their wide applications in food industry, microbiology and laboratorial sciences [2–3]. Even though agar and pure **1** are readily and commercially available, these polysaccharides are far little explored as sources of chemical platforms for drug development. The rare monosaccharide 3,6-anhydro-α-L-galactopyranose (AnGal), naturally present in the agarose structure, is a valuable scaffold since it is a highly functionalized and a chiral-rich monomer. This moiety is indeed presumed to play an important role in the bioactivity of agar-oligosaccharides [4–6].

**Scheme 1:**
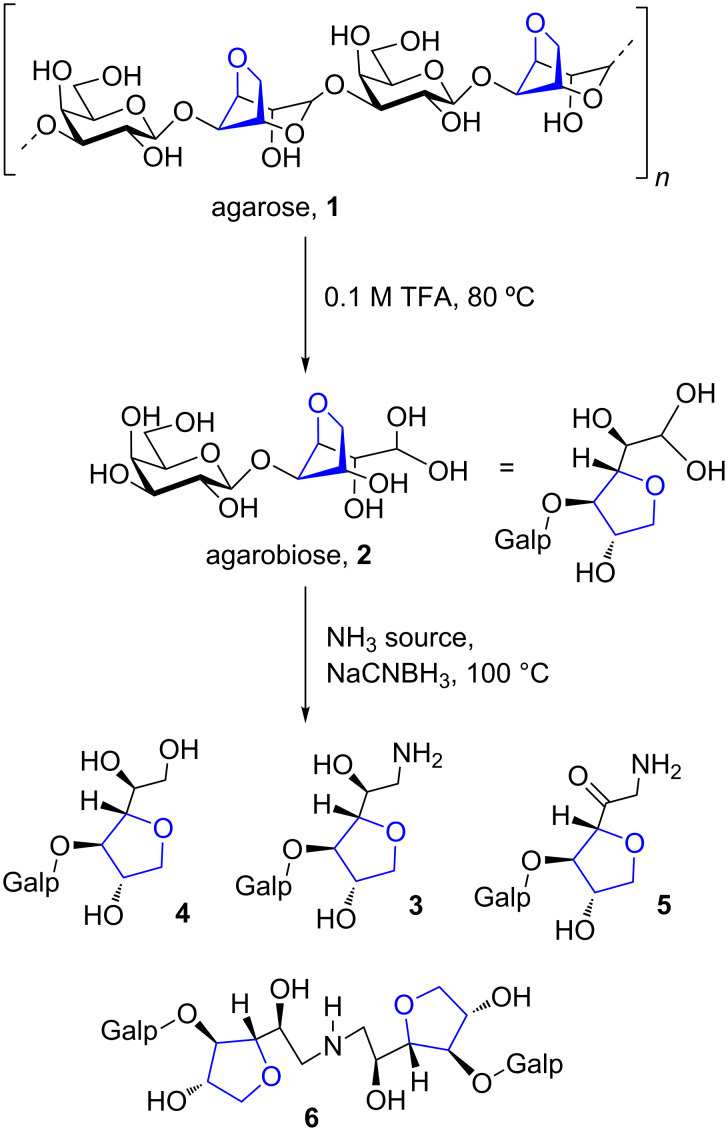
Overview of the hydrolysis–reductive amination procedure to produce primary glycamine **3** and byproducts.

As AnGal appears polymerized within the agarose backbone, a conceivable way to obtain it as a free monosaccharide would involve the cleavage of the glycosidic linkages of the polysaccharide. The unpolymerized product shows AnGal as a *C*-threofuranose motif [7] (see agarobiose, **2**, Scheme 1) and because *C*-glycosides are useful moieties for chemical synthesis and medicinal chemistry, agarose hydrolysis products can be considered interesting carbohydrate-based building blocks [8] to be employed in drug discovery. A part of our group has utilized this type of approach for the synthesis of dihydropyridine glycoconjugates [9] and antiviral sulfated alkylglycosides [10]. In an effort to amplify the utility of AnGal as a starting material for the synthesis of bioactive compounds, we have recently envisaged that its substituted *C*-threofuranose ring could serve as an excellent scaffold to build muscarine analogues [11]. Cholinergic agents targeting muscarinic acetylcholine receptors (mAChRs) have historically served for diverse applications in medicine [12–13] and recently regained interest because of their potential as drug candidates for the treatment of Alzheimer’s disease [14–17].

To effectively generate muscarine analogues from AnGal, an adequate strategy for the placement of an exocyclic amino group attached to the *C*-threofuranose ring is required. Because of the presence of an aldehyde hydrate moiety associated to free AnGal, such a strategy could be conducted through reductive amination reactions. Even though reductive amination has been widely used in organic synthesis, its applicability remains challenging when dealing with carbohydrates [18–20]. Rearrangements, enolization and dimerization are among the drawbacks that can be encountered during the transformation of mono- or oligosaccharides directly into glycamines. Considering (a) the potential of AnGal as starting material for the synthesis of glycamines structurally related to muscarine, (b) the suitability of reductive amination to accomplish such a synthesis through a short protecting-group-free route and (c) the compatibility of the starting material and all reaction conditions to the aqueous media, here we present the aqueous semisynthesis of mono- and disaccharide glycamines obtained by hydrolysis and reductive amination from commercially available agar.

**Scheme 2:**
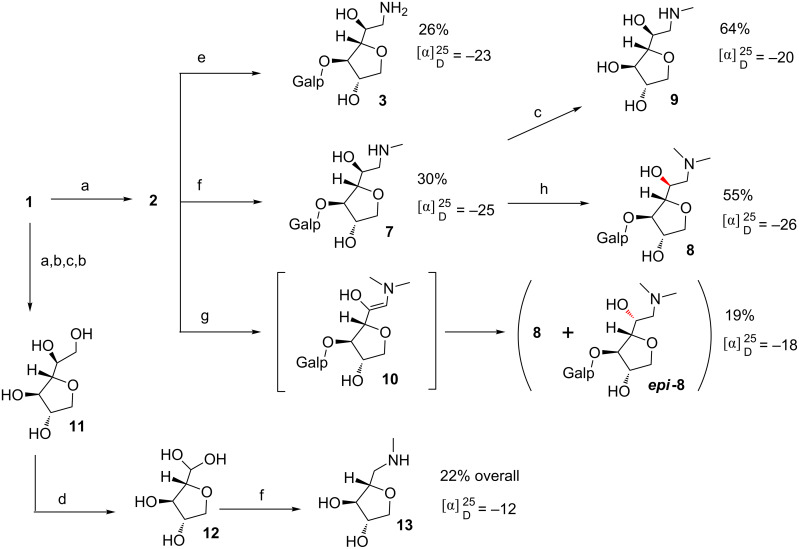
Overview of synthetic procedures, yields and specific rotation of glycamines **3**, **7, 8**, *epi*-**8, 9** and **13**. a) 0.1 M TFA, 80 °C; b) NaBH_4_; c) 2.0 M TFA, 110 °C; d) NaIO_4_; e) NH_4_Ac, NaCNBH_3_, pH 11, 100 °C; f) HCl·NH_2_Me, NaCNBH_3_, pH 11, 100 °C; g) HCl·NHMe_2_, NaCNBH_3_, pH 11, 100 ºC; h) HCHO, NaCNBH_3_, pH 11, 70 °C.

Initially, in order to obtain the appropriate substrate for the synthesis of the *C*-glycoside glycamines **3**, **7** and **8** (Scheme 2), agarose (**1**) was submitted to partial hydrolysis to produce disaccharide agarobiose (**2**, Scheme 1). For this purpose, we implemented a previously described hydrolysis process [8], which was conducted in aqueous TFA for 3 h at 80 °C to give a crude hydrolysate containing **2** as main constituent (for detailed procedures and NMR assignment see Supporting Information File 1). Considering the process of obtaining AnGal or AnGal-containing oligosaccharides from **1** by acid hydrolysis, it is important to notice that the 3,6-anhydrogalactosidic bonds are more acid labile than most of pyranoses. In this way, if AnGal is not produced as a free monosaccharide, it is found as the reducing terminal of the resulting oligosaccharides. Due to torsion angle restraints imposed by the preexisting 3,6-anhydro five-membered ring [21], free AnGal is also not able to recover its original pyranosidic form that is found within the polysaccharide structure. As a consequence, **2** presented a *C*-glycofuranose unit bearing a two-carbon appendix, which presents a hydroxy and an aldehyde hydrate group. Yet obtaining this type of product is not an easy task because of its acid liability. Hydrolysis conditions must be mild to avoid dehydration leading to hydroxymethyl furaldehyde byproduct [22].

The crude agarose hydrolysate containing **2** was then directly employed for the synthesis of the primary glycamine **3** by means of reductive amination, which could be entirely conducted in aqueous media. Glycamines of monosaccharides have been extensively prepared via reductive amination; however, there are just few and laborious examples for the preparation of 1-deoxy-1-aminodisaccharides (those prepared from lactose, maltose and cellobiose) [23]. The primary stage of our optimization to obtain **3** consisted in evaluating the influence of different ammonium salts or ammonium hydroxide as nitrogen source to the reductive amination reactions (Table 1, entries 1–6). During this study we observed common byproducts related to the reductive amination of sugars [19] (Scheme 1). All the reaction conditions tested (Table 1) showed the presence of alditol **4** as a result of sodium cyanoborohydride lack of selectivity [20] to reduce imines in the presence of aldehydes. When using ammonium chloride and ammonium sulfate (Table 1, entries 5 and 6) a distinct product was detected in the reaction medium by mass spectroscopy (see Supporting Information File 1), which probably corresponded to a 1-amino-1-deoxyketose (compound **5**). This is known as an Amadori product [18] obtained from the characteristic rearrangement of the 1-deoxy-2-hydroxyimine intermediate. Yet, the secondary amine *bis*-disaccharide **6** was frequently found as byproduct in the present work. It is a consequence of a sequential reductive amination of **3** with the remaining starting material **2**. Organic ammonium salts, acetate (Table 1, entry 3) and oxalate (Table 1, entry 4), provided superior yields compared with their inorganic counterpart, sulfate and chloride. The reductive amination reactions were herein conducted at pH 11. Although typical reductive aminations are conducted under acidic conditions, the use of basic settings has been advantageous to primary glycamines synthesis [19]. Under basic conditions increased amounts of free-base ammonia are created, thereby favoring the nucleophilic attack at the carbonyl group. Moreover, the base helps deprotonate the iminium ion intermediate pushing the reaction towards the imine product [20]. Attempts to conduct this reaction on acidic conditions failed as in other glycamines synthesis [19]. Experiment outlined in Table 1, entry 1, using ammonium hydroxide as ammonia source resulted in a complex mixture with no evidence of amine **3** formation. Reaction using ammonium carbonate (Table 1, entry 2) also ended up in a messy byproduct mixture and the poor isolated yield was also a consequence of the exhaustive chromatographic cleanup utilized. The most problematic issue was to avoid byproduct **6**. Because of its similar adsorption characteristics to the desired amine **3**, it was difficult to chromatographically eliminate **6**. We could avoid **6** (and boost **3** yields significantly) by increasing the excess of ammonia (Table 1, entry 7) and by diluting the reaction media (Table 1, entry 9). These findings corroborate with the work of Dangerfield and co-workers [19]. Another pivotal aspect to achieve reasonable yields of **3** was the purification procedure. The huge excess of ammonium salts had a dreadful impact on workup since all cleanup steps were performed in aqueous media. The strategy employed to eliminate the salts was based on the use of a large amount of anionic exchange resin, which by eliminating acetate ions enabled the elimination of remaining ammonia by reduced pressure evaporation. The amino sugar rich fraction was then concentrated and chromatographed on silica gel using MeOH/2 M NH_4_OH as mobile phase. An aqueous solution containing the isolated free-base glycamine was then carefully conducted to pH 4.0 with HCl to obtain the respective hydrochloride salt, which gave rise to a good quality NMR spectra.

**Table 1:** Synthesis optimization of glycamine **3**^a^.

Entry	NH_3_ source	NH_3_ (equiv)	Time (h)	[2] (mM)	**4**^b^	**5**^b^	**6**^b^	Yield **3**^c^ (%)

1^d^	NH_4_OH	20	2	150	+	–	–	0
2	(NH_4_)_2_CO_3_	20	2	150	+	–	+	5
3	NH_4_Ac	20	2	150	+	–	+	12
4	(NH_4_)_2_C_2_O_4_	20	2	150	+	–	+	10
5	(NH_4_)_2_SO_4_	20	2	150	+	+	+	7
6	NH_4_Cl	20	2	150	+	+	+	5
7	NH_4_Ac	40	2	150	+	–	+	15
8	NH_4_Ac	40	5	150	+	–	+	18
9	NH_4_Ac	40	5	60	+	–	–	26

^a^All reactions conducted using aqueous media, 100 mg of **2**, sealed tube, 100 °C, 2.0 equiv NaCNBH_3_ and pH 11 adjusted with triethylamine (TEA). ^b^Presence (+) or absence (–) of byproduct **4**, **5** or **6**. Byproducts were detected in the reaction mixtures by TLC and characterized exclusively by low resolution mass spectrometry (LRMS, see Supporting Information File 1). Compound **4**: LRMS *m*/*z* calc. for [M + Na]^+^ C_12_H_22_NaO_10_^+^: 349.1105; found: 349.10. Compound **5**: LRMS *m*/*z* calc. for [M + H]^+^ C_12_H_22_NO_9_^+^: 324.1289; found: 324.19. Compound **6**: LRMS *m*/*z* calc. for [M + H]^+^ C_24_H_44_NO_18_^+^: 634.2553; found: 634.27. ^c^Isolated yields. ^d^pH > 12.

**Figure 1:**
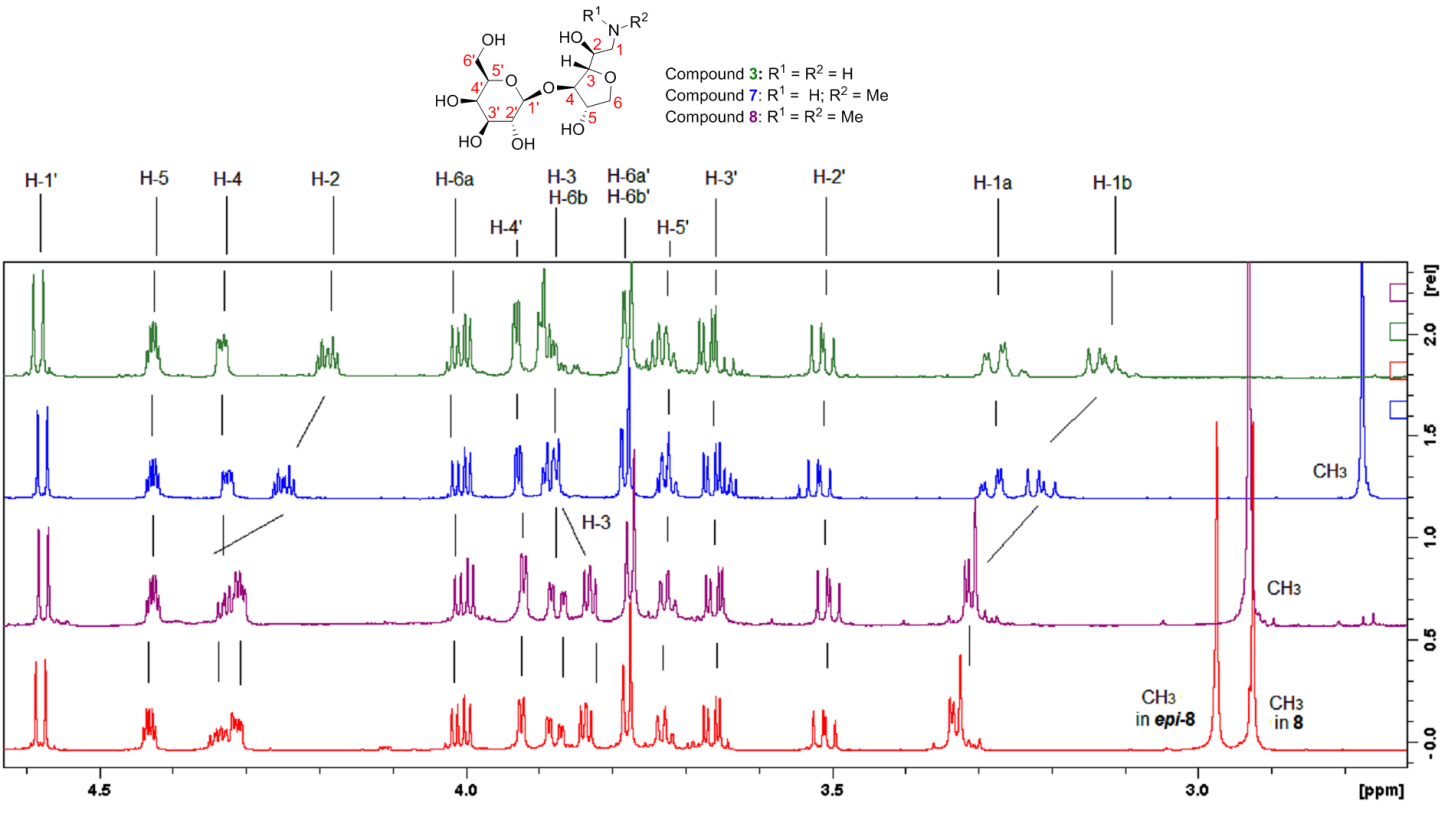
^1^H NMR spectrum comparison of glycamines at 600 MHz, D_2_O, pH 4.0. Compound **3** in green, **7** in blue, **8** in purple and equimolar mixture **8**+*epi-***8** in red.

We then applied the optimal conditions established for the synthesis of **3** (Table 1, entry 9) to obtain the secondary and tertiary glycamines **7** and **8** (Scheme 2). By performing the reductive amination between **2** and dimethylamine hydrochloride we obtained a mixture of epimers (**8** and *epi*-**8**) which differed on the stereochemistry of the C-2 of the 3,6-anhydro amino sugar moiety. This was related to the fact that during the course of the reductive amination, the intermediate to be reduced is enamine **10** (represented in Scheme 2). ^1^H NMR of the mixture of **8** and *epi*-**8** indicated equal amounts for the two epimers, which gave two identical integration values for the distinctive *N*-methyl signals at 2.98 and 2.92 ppm, attributed to *epi*-**8** and **8**, respectively (see Figure 1, spectrum in red). Aside from the *N*-methyl chemical shifts, NMR signals are otherwise indistinguishable for both epimers. This result indicated that even with prochiral C-2 of **10** being surrounded by a covalently linked chiral environment, no internal asymmetric induction took place during the reduction. The overall yields, which ranged between 19–30% for glycamine synthesis (Scheme 2), were considered satisfactory as they were calculated from crude agar starting material. Additionally, we highlight the 22% overall yield in the six-step synthesis of **13** as a good achievement. For detailed synthesis procedures see Supporting Information File 1.

We could access pure **8** by reacting **7** and formaldehyde under reductive conditions. The reaction product **8** kept the original configuration of its parenting analogue, amine **7**, because the reaction between such primary amine **7** and formaldehyde occurs via an imine intermediate (not shown), instead of with a prochiral enamine intermediate (such is the case of **10**). Specific rotation values ([α]_D_) endorsed the above mentioned configuration aspects. Comparison of the ^1^H NMR assignments shown by **3**, **7** and **8** indicated that the extra methyl appendices on amino group had impact on neighborhood chemical shifts (note H-1a, H-1b, H-2 and H-3, Figure 1). Even the coupling constant values (*J*) were slightly influenced (*J*_H2-H3_ = 3.6 Hz in **3** and **7**; *J*_H2-H3_ = 3.9 Hz in **8**, Table 2). The aforementioned might translate small conformational changes.

**Table 2:** ^1^H and ^13^C NMR assignments of the AnGal unit of compounds **3**, **7** and **8**.

Compound	**3**			**7**			**8**	
					
Position	^1^H^a^	^13^C^b^		^1^H^a^	^13^C^b^		^1^H^a^	^13^C^b^

1a	3.27 (dd, 3.6)	42.9		3.28 (dd, 3.6)	51.9		3.33 (m)^nd^	59.7
1b	3.12 (dd, 8.8)	42.9		3.22 (dd, 9.0)	51.9		3.33 (m)^nd^	59.7
2	4.18 (dt,**3.6**/8.8)	67.2		4.24 (dt, **3.6**/9.0)	66.0		4.33 (dt, **3.9**/9.2)	64.9
3	3.87 (m)^nd^	85.0		3.87 (m)^nd^	84.3		3.84 (dd, 3.9/4.9)	84.1
4	4.32 (ddd, 0.5/2.5/4.9)	85.9		4.32 (ddd, 0.5/2.5/4.9)	85.9		4.31 (ddd, 0.5/2.5/4.9)	85.9
5	4.42 (dt, 2.5/4.7)	76.0		4.42 (dt, 2.5/4.7)	76.0		4.42 (dt, 2.5/4.7)	76.0
6a	4.00 (dd, 4.7/10.2)	73.8		4.00 (dd, 4.7/10.2)	73.8		4.00 (dd, 4.7/10.2)	73.8
6b	3.88 (m)^nd^	73.8		3.88 (m)^nd^	73.8		3.88 (dd, 2.5/ 10.2)	73.8
*N*-Me				2.77 (s)	33.7		2.92 (s)	42.0

^a1^H NMR assignment at 600 MHz. Data acquired in D_2_O at pH 4.0. Given values in δ ppm. Multiplicity and coupling constants (*J*) in Hz are given in brackets. ^b13^C NMR chemical shifts determined by HSQC ^1^H,^13^C correlation experiments. ^nd^coupling constant not determined due to signal overlapping.

**Figure 2:**
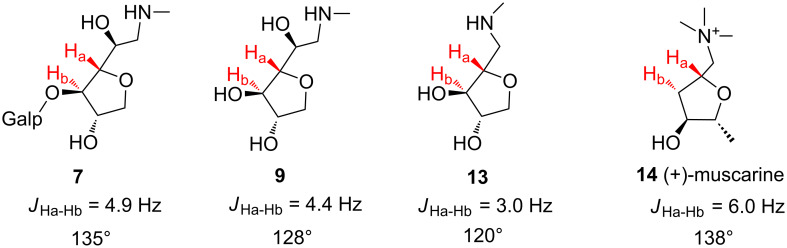
Comparison of the ring distortion among glycamines **7**, **9** and **13**, and (+)-muscarine **14**. Torsion angles calculated with DAD equation [24]. Experimental coupling data from ^1^H NMR at 600 MHz, D_2_O, pH 4.0. Compound **14** NMR coupling data retrieved from literature [25].

In order to extend our library, and to exercise starting material **1** versatility, we synthesized two other derivatives from AnGal, in this case lacking the galactopyranose unit (compounds **9** and **13**, Scheme 2). A handy approach would involve utilizing free AnGal, in its aldehyde form, as starting material; however, to the best of our knowledge, there is no available methodology to obtain this building block using chemical hydrolysis. We produced compound **9** by hydrolysis of the previously synthesized glycamine **7**. In addition, we conducted a stepwise sequence of hydrolysis and reduction reactions, followed by periodate cleavage of the 1,2-vicinal diol of **11** to give **12**. From there, we obtained the corresponding methylamine derivative **13** by using the reductive amination conditions outlined herein. Compound **13** presents one less carbon between the five-membered ring and the amino group, in comparison to the other glycamines obtained. Both aminomonosaccharides **9** and **13** are interesting moieties, regarding their resemblance with the bioactive (+)-muscarine **14** (Figure 2). Indeed, ongoing docking and molecular dynamics experiments revealed the amino-AnGal moiety as a promising platform to launch the design of new mAChR modulators [11]. The differences in *J*_Ha-Hb_ (Figure 2) observed between methylglycamines **7**, **9** and **13** evidenced a ring distortion. The influence of stereochemical features on the biological activity of those and other glycamines will be subject of future publications.

## Conclusion

In sum we have explored agarose backbone and AnGal units to obtain *C*-glycofuranose-containing glycamines through a short, protecting-group-free and aqueous-based semisynthesis. Our present study also addressed the obstacles frequently faced in reductive amination of carbohydrates (such as byproduct accumulation and purification issues) and contributed to provide additional information to access a more efficient synthesis methodology. New amino-substituted *C*-threofuranosides, glycamines **3**, **7**, **8**, *epi-***8**, **9** and **13**, were synthesized and characterized (see below general synthesis procedures and assignments). By adopting an alternative synthetic route, compound **8** could be obtained in the absence of its epimer, *epi*-**8**. Moreover, the chemical entities described herein offer opportunities for studies in glycobiology, biomedicinal chemistry and drug design. The use of these compounds as starting material to access new small ligands as GPCR modulators is ongoing in our laboratories and will be subject of a forthcoming publication.

## Experimental

### Reductive amination general procedure

Carbohydrate aldehyde hydrate (1.5 mmol) was dissolved in water (25 mL) then ammonium salts (40 equiv) or methylamine hydrochloride (30 equiv) or dimethylamine hydrochloride (8.5 equiv) were added and homogenized. pH 11 was adjusted with TEA and finally sodium cyanoborohydride (2 equiv) was added in a single portion. The flask was tightly closed immediately. This mixture was placed in a 100 °C glycerin bath and stirred for 5 hours. Hereafter the media was concentrated under reduced pressure, redissolved in water (100 mL) and stirred with strongly basic anion exchange resin (Amberlite IRA 410 – OH^−^ form, 100 mL, 68 g) for 1 hour. The resin was filtered and washed with water (2 × 100 mL). The filtrate was dried under reduced pressure using co-evaporation with EtOH. This crude material was dissolved in warm MeOH (50 mL), filtered through a borosilicate sintered funnel and dried under reduced pressure. Finally the crude was submitted to flash chromatography (Silica Gel 60, Eluent: MeOH/2 M NH_4_OH 6:1) to give the pure glycamine.

### Physical data and spectral assignments

**Compound 3:** [α]_D_: −23.0 (*c* 1.0, H_2_O); ^1^H NMR (600 MHz, D_2_O, pH 4.0, ppm) δ 4.57 (d, *J*_H1’-H2’_ = 7.8 Hz, 1H, H1’), 4.42 (dt, *J*_H5-H4_ = 2.5 Hz, *J*_H5-H6a_ = 4.7 Hz, 1H, H5), 4.32 (ddd, *J =* 0.5 Hz, *J*_H4-H5_ = 2.5 Hz, *J*_H4-H3_ = 4.9 Hz, 1H, H4), 4.18 (dt, *J*_H2-H3_ = 3.6 Hz, *J*_H2-H1a_ = 8.8 Hz, 1H, H2), 4.00 (dd, *J*_H6a-H5_ = 4.7 Hz, *J*_H6a-H6b_ = 10.2 Hz, 1H, H6a), 3.92 (dd, *J*_H4’-H5’_ = 0.7 Hz, *J*_H4’-H3’_ = 3.4 Hz, 1H, H4’), 3.88 (m, 1H, H6b), 3.87 (m, 1H, H3), 3.78 (dd, *J*_H6’-H5’_ = 5.9 Hz, 2H, H6a’ and H6b’), 3.71 (m, 1H, H5’), 3.66 (dd, *J*_H3’-H4’_ = 3.4 Hz, *J*_H3’-H2’_ = 9.9 Hz, 1H, H3’), 3.51 (dd, *J_H_*_2’-H1’_ = 7.8 Hz, *J*_H2’-H3’_ = 9.9 Hz, 1H, H2’), 3.27 (dd, *J*_H1a-H2_ = 3.6 Hz, 1H, H1a), 3.12 (dd, *J*_H1b-H2_ = 8.8 Hz, 1H, H1b); ^13^C NMR (600 MHz, D_2_O, pH 4.0, ppm) δ 103.0 (C1’), 85.9 (C4), 85.0 (C3), 76.2 (C5’), 76.0 (C5), 73.8 (C6), 73.3 (C3’), 71,4 (C2’), 69.4 (C4’), 67.2 (C2), 62.0 (C6’), 42.9 (C1); HRMS: *m*/*z* [M + H]^+^ calcd for C_12_H_24_NO_9_^+^: 326.1451, found: 326.1469.

**Compound 7:** [α]_D_: −25.0 (*c* 1.0, H_2_O); ^1^H NMR (600 MHz, D_2_O, pH 4.0, ppm) δ 4.57 (d, *J*_H1’-H2’_ = 7.8 Hz, 1H, H1’), 4.42 (dt, *J*_H5-H4_ = 2.5 Hz, *J*_H5-H6a_ = 4.7 Hz, 1H, H5), 4.32 (ddd, *J =* 0.5 Hz, *J*_H4-H5_ = 2.5 Hz, *J*_H4-H3_ = 4.9 Hz, 1H, H4), 4.24 (dt, *J*_H2-H3_ = 3.6 Hz, *J*_H2-H1a_ = 9.0 Hz, 1H, H2), 4.00 (dd, *J*_H6a-H5_ = 4.7 Hz, *J*_H6a-H6b_ = 10.2 Hz, 1H, H6a), 3.92 (dd, *J*_H4’-H5’_ = 0.7 Hz, *J*_H4’-H3’_ = 3.4 Hz, 1H, H4’), 3.88 (m, 1H, H6b), 3.87 (m, 1H, H3), 3.78 (dd, *J*_H6’-H5’_ = 5.9 Hz, 2H, H6a-b’), 3.71 (m, 1H, H5’), 3.66 (dd, *J*_H3’-H4’_ = 3.4 Hz, *J*_H3’-H2’_ = 9.9 Hz, 1H, H3’), 3.51 (dd, *J*_H2’-H1’_ = 7.8 Hz, *J*_H2’-H3’_ = 9.9 Hz, 1H, H2’), 3.28 (dd, *J*_H1a-H2_ = 3.6 Hz, 1H, H1a), 3.22 (dd, *J*_H1b-H2_ = 9.0 Hz, 1H, H1b), 2.77 (s, 1H, CH_3_); ^13^C NMR (600 MHz, D_2_O, pH 4.0, ppm) δ 103.0 (C1’), 85.9 (C4), 84.3 (C3), 76.2 (C5’), 76.0 (C5), 73.8 (C6), 73.3 (C3’), 71,4 (C2’), 69.4 (C4’), 66.0 (C2), 62.0 (C6’), 51.9 (C1), 33.7 (CH_3_); HRMS: *m*/*z* [M + H]^+^ calcd for C_13_H_26_NO_9_^+^: 340.1608, found: 340.1631.

**Compound 8 + *epi*-8:** [α]_D_: −26.0 (*c* 1.0, H_2_O) for pure compound **8***;* [α]_D_: −18.0 (*c* 1.0, H_2_O) for equimolar mixture (**8** + *epi*-**8**); ^1^H NMR (600 MHz, D_2_O, pH 4.0, ppm) δ 4.57 (d, *J*_H1’-H2’_ = 7.8 Hz, 1H, H1’), 4.42 (dt, *J*_H5-H4_ = 2.5 Hz, *J*_H5-H6a_ = 4.7 Hz, 1H, H5), 4.33 (dt, *J*_H2-H3_ = 3.9 Hz, *J*_H2-H1a_ = 9.2 Hz, 1H, H2), 4.31 (ddd, *J =* 0.5 Hz, *J*_H4-H5_ = 2.5 Hz, *J*_H4-H3_ = 4.9 Hz, 1H, H4), 4.00 (dd, *J*_H6a-H5_ = 4.7 Hz, *J*_H6a-H6b_ = 10.2 Hz, 1H, H6a), 3.92 (dd, *J*_H4’-H5’_ = 0.7 Hz, *J*_H4’-H3’_ = 3.4 Hz, 1H, H4’), 3.88 (dd, *J*_H6b-H5_ = 2.5, *J*_H6b-H6a_ = 10.2, 1H, H6b), 3.84 (dd, *J*_H3-H4_ = 4.9 Hz, 1H, H3), 3.78 (dd, *J*_H6’-H5’_ = 5.9 Hz, 2H, H6a’/6b’), 3.71 (m, 1H, H5’), 3.66 (dd, *J*_H3’-H4’_ = 3.4 Hz, *J*_H3’-H2’_ = 9.9 Hz, 1H, H3’), 3.51 (dd, *J*_H2’-H1’_ = 7.8 Hz, *J*_H2’-H3’_ = 9.9 Hz, 1H, H2’), 3.33 (m, 2H, H1a and H1b), 2.98 (s, *epi*-**8** CH_3_), 2.92 (s, compound **8** CH_3_); ^13^C NMR (600 MHz, D_2_O, pH 4.0, ppm) δ 103.0 (C1’), 85.9 (C4), 84.1 (C3), 76.2 (C5’), 76.0 (C5), 73.8 (C6), 73.3 (C3’), 71,4 (C2’), 69.4 (C4’), 64.9 (C2), 62.0 (C6’), 59.7 (C1), 45.0 (*epi*-**8** CH_3_), 42.0 (compound **8** CH_3_); HRMS: *m*/*z* [M + H]^+^ calcd for C_14_H_28_NO_9_^+^: 354.1764, found: 354.1764.

**Compound 9:** [α]_D_: −20.0 (c 1.0, H_2_O); ^1^H NMR (400 MHz, D_2_O) δ (ppm) 4.25 (m, *J =* 2.6 Hz, 1H, H5), 4.13 (m, 2H, H2, H4), 4.01 (dd, *J =* 2.6/9.9, 1H, H6a), 3.85 (dd, *J =* 9.9, 1H, H6b), 3.73 (m, *J*_H3-H4_ = 4.4 Hz, 1H, H3), 3.27 (dd, *J =* 12.9 Hz, 1H, H1a), 3.15 (m, 1H, H1b), 2.77 (s, 3H, CH_3_); ^13^C NMR (400 MHz, D_2_O) δ (ppm) 86.0 (C3), 78.7 (C4), 77.5 (C5), 73.5 (C6), 66.7 (C2), 52.1 (C1), 33.5 (CH_3_); HRMS: *m*/*z* [M + H]^+^ calcd for C_7_H_16_NO_4_^+^: 178.1079, found: 178.1072.

**Compound 13:** [α]_D_: −12.0 (*c* 1.0, H_2_O); ^1^H NMR (400 MHz, D_2_O, ppm) δ 4.18 (dd, *J* = 1.6/3.6 Hz, 1H, H4), 3.97 (m, 2H, H5a and H3), 3.95 (m, *J*_H2-H3_ = 3.0 Hz, 1H, H2), 3.84 (dd, *J =* 1.6/10.1 Hz, H5b), 3.08 (m, 2H, H1a and H1b), 2.57 (CH_3_); ^13^C NMR (400 MHz, D_2_O, ppm) δ 81.7 (C-2), 79.1 (C-3), 76.5 (C-4), 73.2 (C-5), 50.9 (C-1), 33.5 (CH_3_); HRMS: *m*/*z* [M + H]^+^ calcd for C_6_H_14_NO_3_^+^: 148.0974, found: 148.0920.

## Supporting Information

File 1 General methods; LRMS of a reductive amination reaction mixture using NH_4_Cl as NH_3_ source; detailed synthesis procedures; complete assignments and NMR data (^1^H NMR, ^13^C NMR and HSQC spectrum copies).
