# Supplementary material for: Aqueous semisynthesis of C-glycoside glycamines from agarose
Source: Beilstein J Org Chem. 2017 Jun 23;13:1222–9. doi: 10.3762/bjoc.13.121 (PMC5496579; doi:10.3762/bjoc.13.121)
Supplement: File 1 — General methods; LRMS of a reductive amination reaction mixture using NH4Cl as NH3 source; detailed synthesis procedures; complete assignments and NMR data (1H NMR, 13C NMR and HSQC spectrum copies). [file Beilstein_J_Org_Chem-13-1222-s001.pdf]

Supporting information

for

Aqueous semisynthesis of *C*-glycoside glycamines from agarose

Juliana C. Cunico Dallagnol[1], Alexandre Orsato[2], Diogo R. B. Ducatti[3], Miguel D. Noseda[3], Maria Eugênia R. Duarte[3] and Alan G. Gonçalves*[,1]

Address: [1]Departamento de Farmácia, Universidade Federal do Paraná, Avenida Lothario Meissner, 3400, Curitiba, Paraná, Brazil, [2]Departamento de Química, Universidade Estadual de Londrina, Rodovia Celso Garcia Cid (Pr 445), Km 380, PO Box 10011, Londrina, Paraná, Brazil and [3]Departamento de Bioquímica e Biologia Molecular, Universidade Federal do Paraná, Avenida Francisco H. dos Santos, 120, PO Box 19046, Curitiba, Paraná, Brazil

Email: Alan G. Gonçalves - alan.goncalves@ufpr.br

*Corresponding author.

General methods; LRMS of a reductive amination reaction mixture using NH_4Cl as NH_3 source; detailed synthesis procedures; complete assignments and NMR data (^{1}H NMR, ^{13}C NMR and HSQC spectrum copies)

Contents

General methods S2

Low resolution mass spectroscopy of a reductive amination reaction mixture using NH_4Cl as NH_3 source. S3

β-D-Galactopyranosyl-(1'→4)-3,6-anhydro-α-L-galactitol (**2**, agarobiose) S4

β-D-Galactopyranosyl-(1'→4)-1-deoxy-1-amino-3,6-anhydro-α-L-galactitol (**3**) S5

β-D-Galactopyranosyl-(1'→4)-1-deoxy-1-(*N*-methylamino)-3,6-anhydro-α-L-galactitol (**7**) S7

β-D-Galactopyranosyl-(1'→4)-1-deoxy-1-(*N*,*N*-dimethylamino)-3,6-anhydro-α-L-galactitol (**8**)... S9

1-Deoxy-1-(*N*-methylamino)-3,6-anhydro-α-L-galactitol (**9**) S12

3,6-Anhydro-L-galactitol (**11**) S14

2,5-Anhydro-L-lyxitol (**12**) S16

1-Deoxy-1-(*N*-methylamino)-2,5-anhydro-L-lyxitol (**13**) S18

Complete table of NMR assignments for compounds **3**,**7** and **8**. S20

General methods

All reagents and solvents were of reagent grade. Agar, Type A, was purchased from Sigma. Reactions were monitored by thin layer chromatography (TLC) on commercially available pre-coated aluminium-backed plates (Merck Kieselgel 60 F_{254}).Visualization was carried by staining using a solution of ninhydrin (5% in ethanol) or resorcinol (2% in ethanol/H_2SO_4 9:1) to visualize amines or sugars, respectively. Column chromatography was performed with the indicated eluents using Fluka silica gel 60 (particle size 220–440 mesh). Yields refer to chromatographically and spectroscopically pure compounds. For final amines the pH of the NMR samples was adjusted to 4.0 using 1 M HCl aqueous solution. All samples were lyophilized before analysis and were prepared with deuterium oxide (D_2O). ^{1}H NMR, ^{13}C NMR and, HSQC ^{1}H-^{13}C) were obtained with a Bruker Avance DRX 400 or Bruker Avance 600 spectrometer (as indicated) operating at 400,1 MHz or 600,1 MHz, respectively for ^{1}H and 100,63 MHz for ^{13}C. ^{13}C NMR chemical shifts were determined by HSQC ^{1}H,^{13}C correlation experiments. Chemical shifts (δ) were expressed in parts per million (ppm) and coupling constants (*J*) in Hertz (Hz) using the residual solvent peaks (H_2O, δ 4,79) as internal standards. Multiplicities were described as singlet (s), doublet (d), triplet (t), doublet of doublets (dd), doublet of triplets (dt), broad (br), and multiplet (m). Mass spectra were acquired in positive mode using an ESI ion source Walters Micromass Quattro LC–MS/MS for low resolution mass spectra (LRMS) and Bruker Micro TOF-Q II XL for high resolution mass spectra (HRMS) using sodium formate solution as reference. Optical rotation values were obtained with a Jasco P-200 polarimeter equipped with a sodium light source. Specific rotation ($[\alpha]_D$) of 1% (g/mL) compounds solutions in distilled water was calculated at 25 °C.

Low resolution mass spectroscopy of a reductive amination reaction mixture using NH_4Cl as NH_3 source

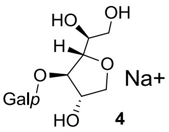

4

Chemical Formula: $C_{12}H_{22}NaO_{10}^{+}$
Exact Mass: 349,1105

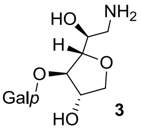

3

Chemical Formula: $C_{12}H_{24}NO_9^{+}$
Exact Mass: 326,1446

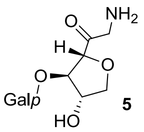

5

Chemical Formula: $C_{12}H_{22}NO_9^{+}$
Exact Mass: 324,1289

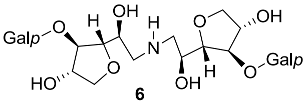

6

Chemical Formula: $C_{24}H_{44}NO_{18}^{+}$
Exact Mass: 634,2553

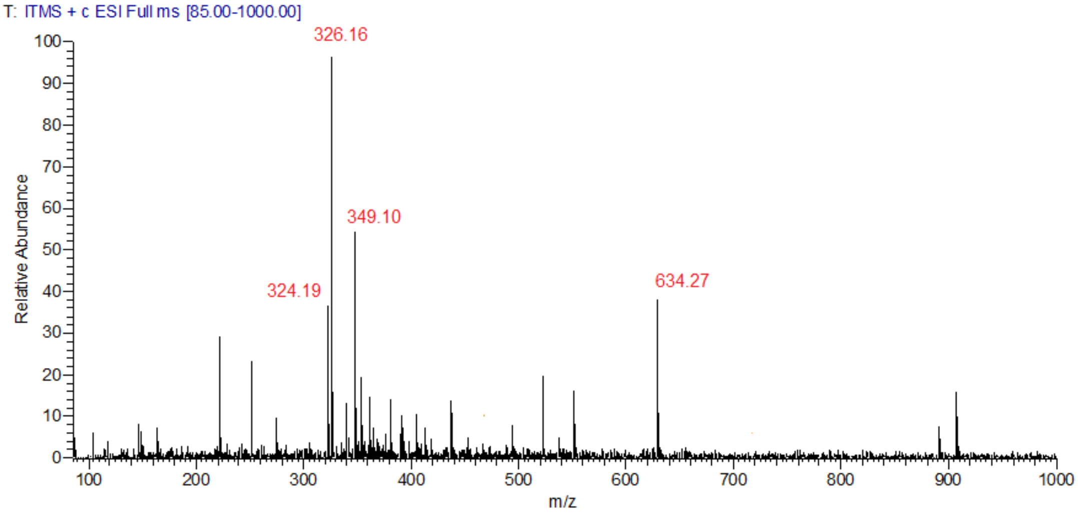

β-D-Galactopyranosyl-(1'→4)-3,6-anhydro- α-L-galactitol (**2**, agarobiose)

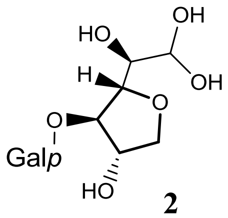

2

Synthesis

Commercial agar (5 g) was first dissolved in hot (90 °C) water (450 mL) and then 1 M TFA aqueous solution (50 mL) was added in one portion to make a final concentration of 0.1 M TFA. The resulting mixture was heated at 80 °C for 3 h, cooled to room temperature, diluted with water (500 mL), and then concentrated under vacuum. The resulting residue was dissolved in water (90 mL), diluted with iPrOH (90 mL), and then filtered through a glass-sintered filter. The filtrate was concentrated and coevaporated with methanol and lyophilized, resulting a light yellow powder as crude material (4.8 g, 96% yield w/w). Material assigned using literature reference.

^{13}C NMS (200 MHz, D_2O) δ (ppm) 102.4 (C1'), 89.9 (C1), 85.4 (C4), 82.8 (C3), 75.2 (C5'), 75.1 (C5), 73.0 (C6), 72.5 (C3'), 72.5 (C2), 70.5 (C2'), 68.6 (C4'), 60.9 (C6'). **HRMS:** m/z calc. for $[M+K]^+$ $C_{12}H_{22}O_{11}K^+$: 381.0799; found: 381.0961. For further characterization data refer to Ducatti, et al, 2011.

Ducatti, DRB, Colodi, FG, Gonçalves, AG, Duarte, MER, Noseda, MD. Production of oligosaccharides by partial acid hydrolysis of galactans. *Brazilian J Phamacogn*, **2011**, 21(2), 296-304.

*Spectral Data of **2***

*13C NMR spectra of **2***

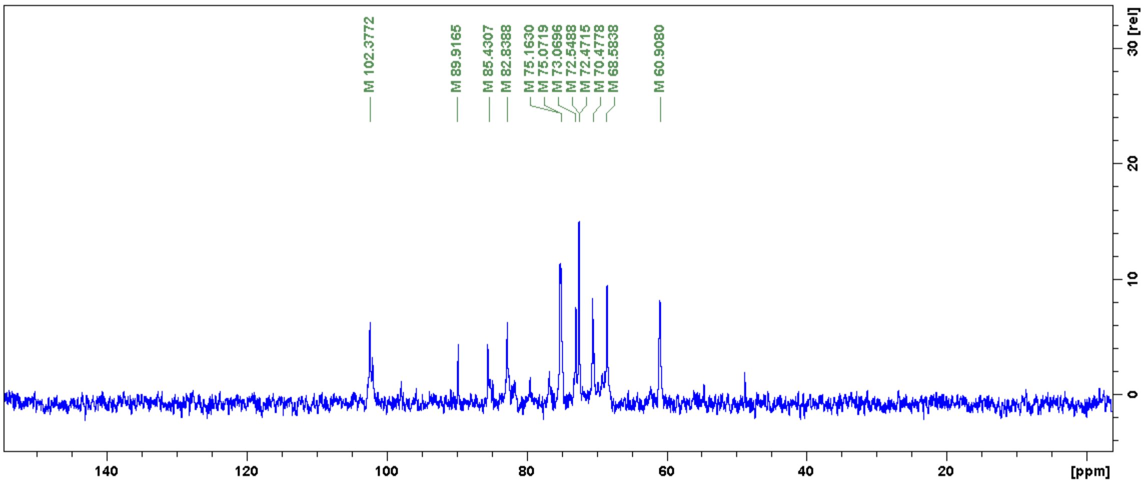

* 100 MHz, D_2O.

β-D-Galactopyranosyl-(1'→4)-1-deoxy-1-amino-3,6-anhydro-α-L-galactitol (**3**)

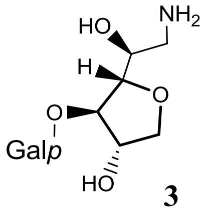

3

Synthesis

Crude material containing agarobiose **2** (500 mg, 1.5 mmol) was dissolved in water (20 mL) then ammonium salts or ammonium hydroxide (40 equiv) was added and homogenized. pH 11 was adjusted with TEA and finally sodium cyanoborohydride (2 equiv, 3.0 mmol, 200 mg) was added in a single portion. The flask was tightly closed immediately. This mixture was placed in a 100 °C glycerin bath and stirred for 5 hours. Hereafter the media was concentrated under reduced pressure, redissolved in water (100 mL) and stirred with strongly basic anion exchange resin (Amberlite IRA 410 - OH^- form, 100 mL, 68 g) for 1 hour. The resin was filtered and washed with water (2 × 100 mL). The filtrate was dried under reduced pressure using coevaporation with EtOH to give a yellow crude product (≈550 mg). Then this material was dissolved in warm MeOH (50 mL), filtered through a borosilicate sintered funnel and dried under reduced pressure, resulting in a vibrant yellow-greenish crude product (≈330 mg). Finally the crude material was submitted to silica gel flash chromatography (eluent: MeOH/2M NH_4OH 6:1) to give the pure aminoglycoside **3** as a white solid after beenig lyophilized (142 mg, 26% molar yield).

Physical and spectral assignment

$[\alpha]_D$: -23.0 (c 1.0, H_2O). **^{1}H NMR** (600 MHz, D_2O, pH 4.0) δ (ppm) 4.57 (d, $J_{H1'-H2'}$ = 7.8 Hz, 1H, H1'), 4.42 (dt, J_{H5-H4} = 2.5 Hz, J_{H5-H6a} = 4.7 Hz, 1H, H5), 4.32 (ddd, J = 0.5 Hz, J_{H4-H5} = 2.5 Hz, J_{H4-H3} = 4.9 Hz, 1H, H4), 4.18 (dt, J_{H2-H3} = 3.6 Hz, J_{H2-H1a} = 8.8 Hz, 1H, H2), 4.00 (dd, J_{H6a-H5} = 4.7 Hz, $J_{H6a-H6b}$ = 10.2 Hz, 1H, H6a), 3.92 (dd, $J_{H4'-H5'}$ = 0.7 Hz, $J_{H4'-H3'}$ = 3.4 Hz, 1H, H4'), 3.88 (m, 1H, H6b), 3.87 (m, 1H, H3), 3.78 (dd, $J_{H6'-H5'}$ = 5.9 Hz, 2H, H6a' and H6b'), 3.71 (m, 1H, H5'), 3.66 (dd, $J_{H3'-H4'}$ = 3.4 Hz, $J_{H3'-H2'}$ = 9.9 Hz, 1H, H3'), 3.51 (dd, $J_{H2'-H1'}$ = 7.8 Hz, $J_{H2'-H3'}$ = 9.9 Hz, 1H, H2'), 3.27 (dd, J_{H1a-H2} = 3.6 Hz, 1H, H1a), 3.12 (dd, J_{H1b-H2} = 8.8 Hz, 1H, H1b). **^{13}C NMR** (600 MHz, D_2O, pH 4.0) δ (ppm) 103.0 (C1'), 85.9 (C4), 85.0 (C3), 76.2 (C5'), 76.0 (C5), 73.8 (C6), 73.3 (C3'), 71,4 (C2'), 69.4 (C4'), 67.2 (C2), 62.0 (C6'), 42.9 (C1). **HRMS**: m/z calc. for $[M+H]^+$ $C_{12}H_{24}NO_9^+$: 326.1451; found: 326.1469.

*Spectral data of **3***

*^{1}H NMR spectra of **3***

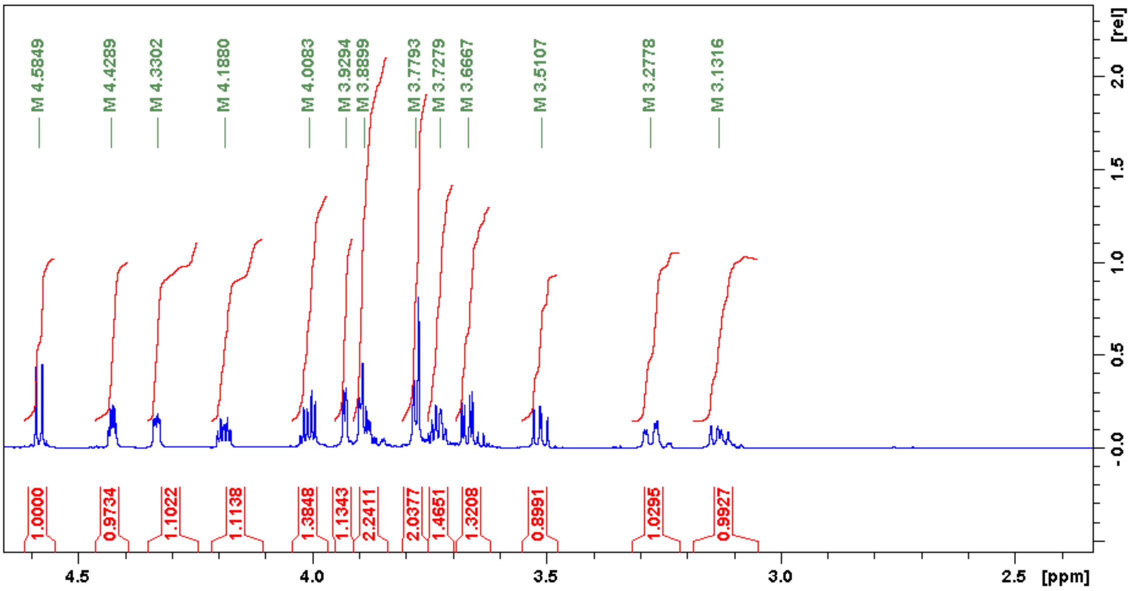

* 600 MHz, D_2O, pH 4.0

*HSQC ^{1}H,^{13}C correlation map of **3***

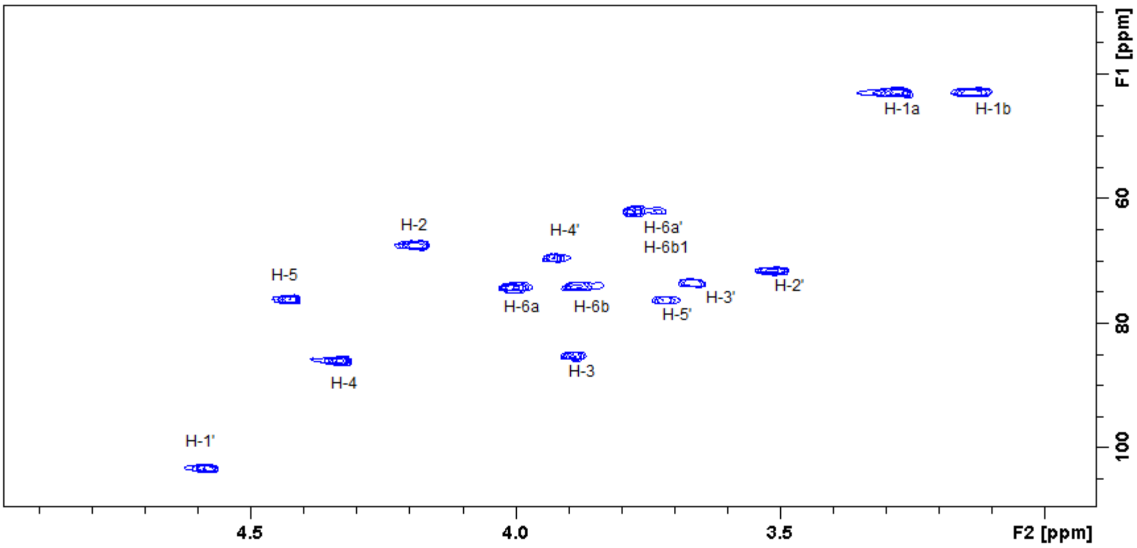

* 600 MHz, D_2O, pH 4.0

β-D-Galactopyranosyl-(1'→4)-1-deoxy-1-(*N*-methylamino)-3,6-anhydro-α-L-galactitol (**7**)

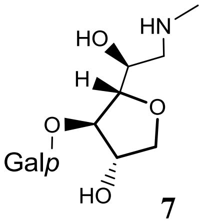

7

Synthesis

Crude material containing agarobiose **2** (500 mg, 1.5 mmol) was dissolved in water (20 mL) then methylamine hydrochloride (20 equiv, 30 mmol, 2.0 g) was added and homogenized. pH 11 was adjusted with TEA and finally sodium cyanoborohydride (2 equiv, 3.0 mmol, 200 mg) was added in a single portion. The flask was tightly closed immediately. This mixture was placed in a 100 °C glycerin bath and stirred for 5 hours. Reaction workup and purification were done as for the primary aminoglycoside **3** synthesis. After chromatography the compound was obtained as a white solid after lyophilization (150 mg, 30% molar yield).

Physical and spectral assignment

[α]$_D$: -25.0 (c 1.0, H_2O). **^{1}H NMR** (600 MHz, D_2O, pH 4.0) δ (ppm) 4.57 (d, $J_{H1'-H2'}$ = 7.8 Hz, 1H, H1'), 4.42 (dt, J_{H5-H4} = 2.5 Hz, J_{H5-H6a} = 4.7 Hz, 1H, H5), 4.32 (ddd, J = 0.5 Hz, J_{H4-H5} = 2.5 Hz, J_{H4-H3} = 4.9 Hz, 1H, H4), 4.24 (dt, J_{H2-H3} = 3.6 Hz, J_{H2-H1a} = 9.0 Hz, 1H, H2), 4.00 (dd, J_{H6a-H5} = 4.7 Hz, $J_{H6a-H6b}$ = 10.2 Hz, 1H, H6a), 3.92 (dd, $J_{H4'-H5'}$ = 0.7 Hz, $J_{H4'-H3'}$ = 3.4 Hz, 1H, H4'), 3.88 (m, 1H, H6b), 3.87 (m, 1H, H3), 3.78 (dd, $J_{H6'-H5'}$ = 5.9 Hz, 2H, H6a-b'), 3.71 (m, 1H, H5'), 3.66 (dd, $J_{H3'-H4'}$ = 3.4 Hz, $J_{H3'-H2'}$ = 9.9 Hz, 1H, H3'), 3.51 (dd, $J_{H2'-H1'}$ = 7.8 Hz, $J_{H2'-H3'}$ = 9.9 Hz, 1H, H2'), 3.28 (dd, J_{H1a-H2} = 3.6 Hz, 1H, H1a), 3.22 (dd, J_{H1b-H2} = 9.0 Hz, 1H, H1b), 2.77 (s, 1H, CH_3). **^{13}C NMR** (600 MHz, D_2O, pH 4.0) δ (ppm) 103.0 (C1'), 85.9 (C4), 84.3 (C3), 76.2 (C5'), 76.0 (C5), 73.8 (C6), 73.3 (C3'), 71,4 (C2'), 69.4 (C4'), 66.0 (C2), 62.0 (C6'), 51.9 (C1), 33.7 (CH_3). **HRMS:** m/z calc. for $[M+H]^+$ $C_{13}H_{26}NO_9^+$: 340.1608; found: 340.1631.

*Spectral data of **7***

*^{1}H NMR spectra of **7***

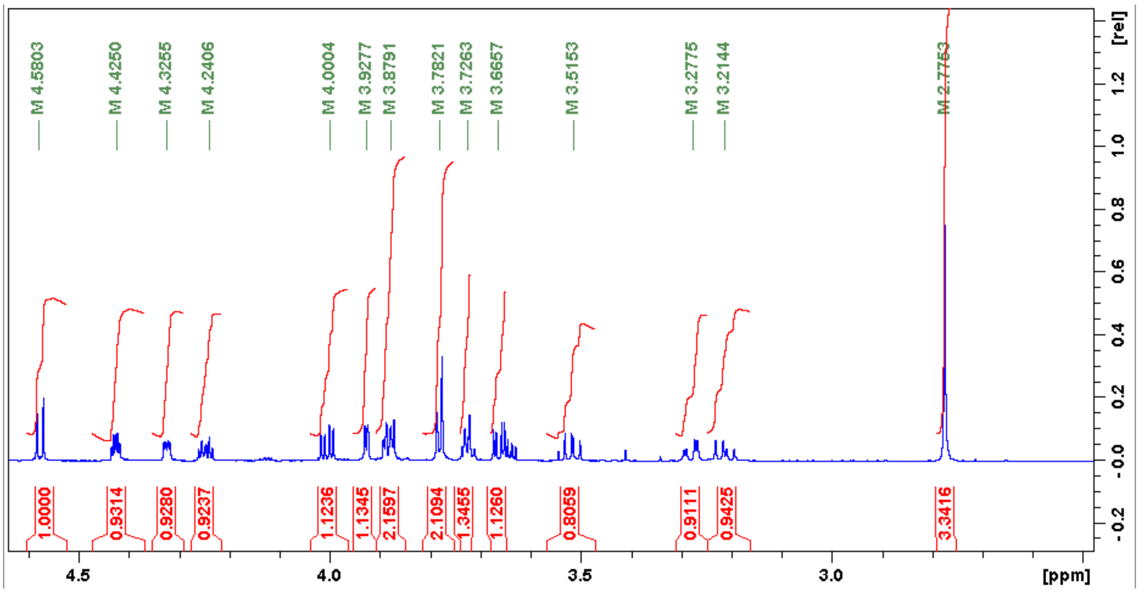

* 600 MHz, D_2O, pH 4.0

HSQC ^{1}H-^{13}C correlation map

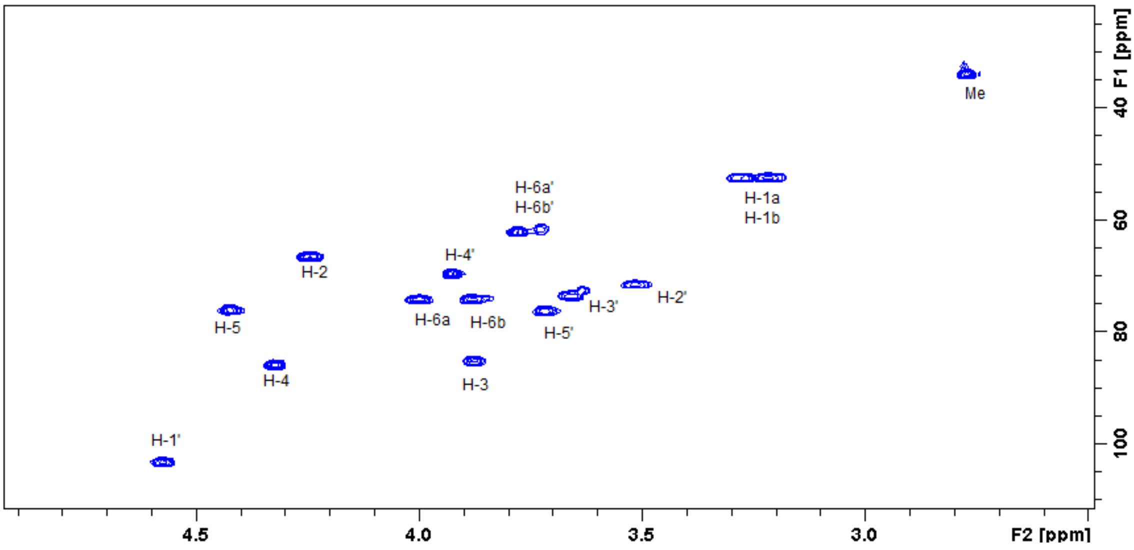

* 600 MHz, D_2O, pH 4.0

β-D-Galactopyranosyl-(1’→4)-1-deoxy-1-(*N*,*N*-dimethylamino)-3,6-anhydro-α-L-galactitol (**8**)

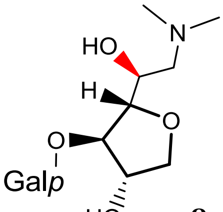

8

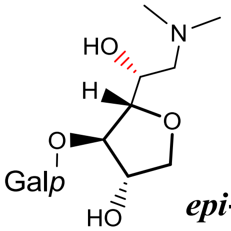

***epi*-8**

Synthesis

Method A - equimolar mixture **8** + **epi-8**

Crude material containing agarobiose **2** (500 mg, 1.5 mmol) was dissolved in water (20 mL) then dimethylamine hydrochloride (8.5 equiv, 13 mmol, 1.0 g) was added and homogenized. pH 11 was adjusted with TEA and finally sodium cyanoborohydride (2 equiv, 3.0 mmol, 200 mg) was added in a single portion. The flask was tightly closed immediately. This mixture was placed in a 100 °C glycerin bath and stirred for 5 hours. Reaction workup and purification were done as for the primary aminoglycoside **3** synthesis. After chromatography an unresolved mixture of **8** + ***epi*-8** showed as a white solid after lyophilization (100 mg, 19% molar yield).

Method B – pure **8**

Methylaminoglycoside **7** (40 mg, 0.12 mmol) was dissolved in water (2 mL) then formaldehyde (36% aqueous solution, 3.0 equiv, 0.36 mmol, 30 µL) was added and homogenized. pH 11 was adjusted with TEA and finally sodium cyanoborohydride (2 equiv, 0.24 mmol, 15 mg) was added in a single portion. The flask was tightly closed immediately. This mixture was placed in a 70 °C glycerin bath and stirred for 5 hours. Reaction workup and purification were done as for the primary aminoglycoside **3** synthesis. After lyophilization, compound **8** was obtained as a white solid (23 mg, 55% molar yield, 13% from agarobiose).

Physical and spectral assignment

Compound 8. **[α]$_D$:** -26.0 (c 1.0, H_2O) for pure compound **8**; **[α]$_D$:** -18.0 (c 1.0, H_2O) for equimolar mixture (**8** + ***epi*-8**). **^{1}H NMR** (600 MHz, D_2O, pH 4.0) δ (ppm) 4.57 (d, $J_{H1'-H2'}$ = 7.8 Hz, 1H, H1'), 4.42 (dt, J_{H5-H4} = 2.5 Hz, J_{H5-H6a} = 4.7 Hz, 1H, H5), 4.33 (dt, J_{H2-H3} = 3.9 Hz, J_{H2-H1a} = 9.2 Hz, 1H, H2), 4.31 (ddd, J = 0.5 Hz, J_{H4-H5} = 2.5 Hz, J_{H4-H3} = 4.9 Hz, 1H, H4), 4.00 (dd, J_{H6a-H5} = 4.7 Hz, $J_{H6a-H6b}$ = 10.2 Hz, 1H, H6a), 3.92 (dd, $J_{H4'-H5'}$ = 0.7 Hz, $J_{H4'-H3'}$ = 3.4 Hz, 1H, H4'), 3.88 (dd, J_{H6b-H5} = 2.5, $J_{H6b-H6a}$ = 10.2, 1H, H6b), 3.84 (dd, J_{H3-H4} = 4.9 Hz, 1H, H3), 3.78 (dd, $J_{H6'-H5'}$ = 5.9 Hz, 2H, H6a'/6b'), 3.71 (m, 1H, H5'), 3.66 (dd, $J_{H3'-H4'}$ = 3.4 Hz, $J_{H3'-H2'}$ = 9.9 Hz, 1H, H3'), 3.51 (dd, $J_{H2'-H1'}$ = 7.8 Hz, $J_{H2'-H3'}$ = 9.9 Hz, 1H, H2'), 3.33 (m, 2H, H1a and H1b), 2.98 (s, ***epi*-8** CH_3), 2.92 (s, compound **8** CH_3). **^{13}C NMR** (600 MHz, D_2O, pH 4.0) δ (ppm) 103.0 (C1'), 85.9 (C4), 84.1 (C3), 76.2 (C5'), 76.0 (C5), 73.8 (C6), 73.3 (C3'), 71,4 (C2'), 69.4 (C4'), 64.9 (C2), 62.0 (C6'), 59.7 (C1), 45.0 (***epi*-8** CH_3), 42.0 (compound **8** CH_3). **HRMS**: m/z calc. for $[M+H]^+$ $C_{14}H_{28}NO_9^+$: 354.1764; found: 354.1764.

*Spectral data (**8** and **epi-8**)*

*^{1}H NMR spectra of pure **8** and **8** + **epi-8***

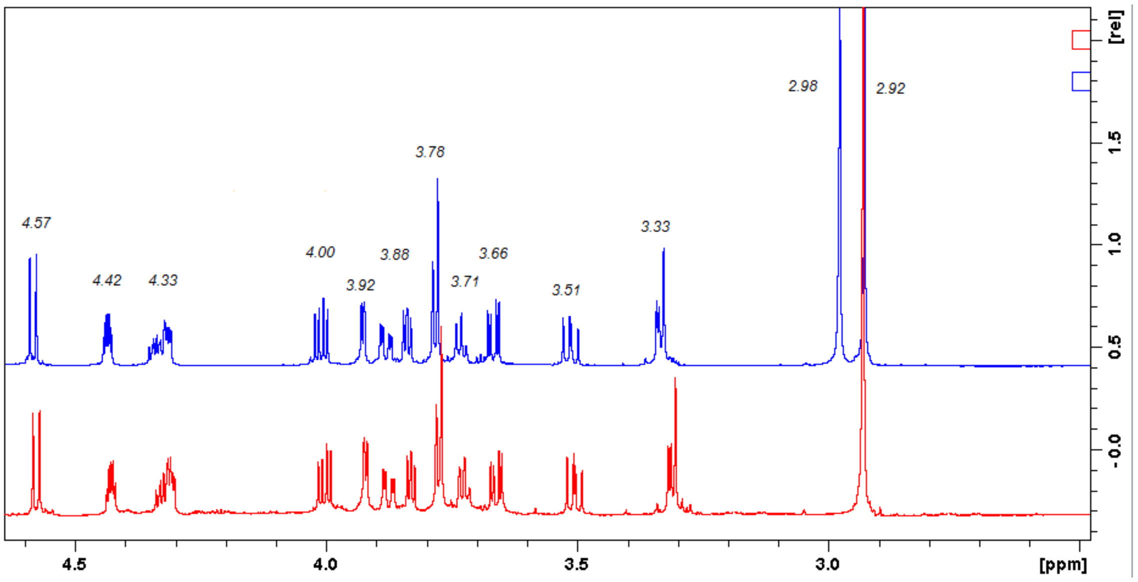

* 600 MHz, D_2O, pH 4.0. Pure compound **8** in red and equimolar mixture of **8** and ***epi*-8** in blue.

*HSQC ^{1}H-^{13}C correlation map equimolar mixture, **8** + **epi-8***

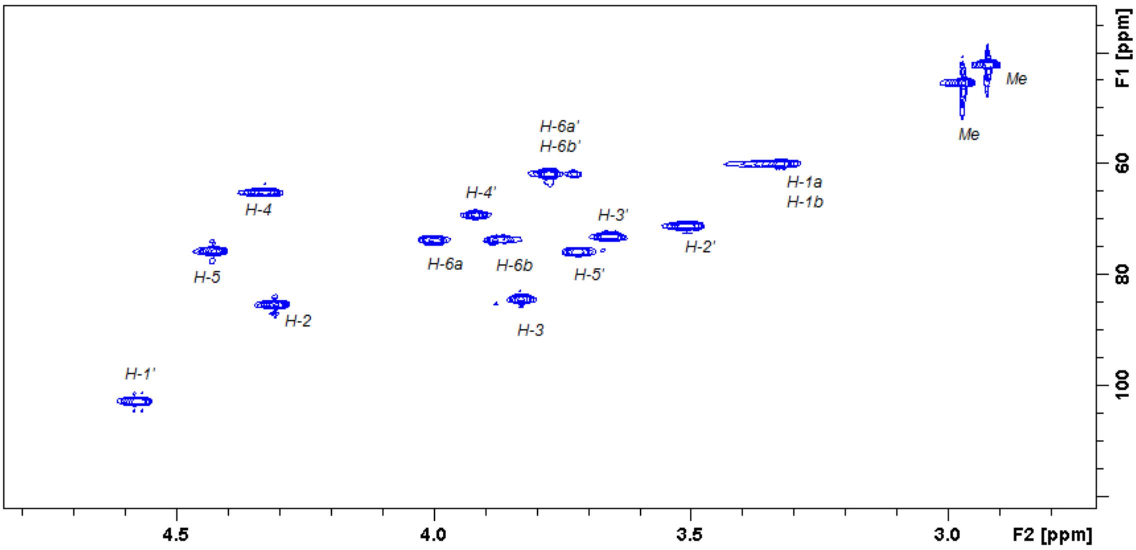

* 600 MHz, D_2O, pH 4.0

1-Deoxy-1-(*N*-methylamino)-3,6-anhydro-α-L-galactitol (**9**)

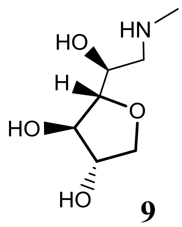

9

Synthesis

Amino disaccharide **7** (100 mg, 0.3 mmol) was dissolved in a 2 M TFA solution (20 mL) and the resulting mixture was heated at 110 °C for 3 h. Then the reaction was cooled to room temperature, diluted with H_2O (20 mL) and then concentrated. The resulting residue was coevaporated with methanol three times to give a syrup. Finally the crude was submitted to flash chromatography (mobile phase: MeOH/2 M NH_4OH 6:1) to give the pure aminoglycoside **9** as a white solid after lyophilized (33 mg, 64% molar yield).

Physical and spectral assignment

$[\alpha]_D$: -20.0 (c 1.0, H_2O). **^{1}H NMR** (400 MHz, D_2O) δ (ppm) 4.25 (m, *J* = 2.6 Hz, 1H, H5), 4.13 (m, 2H, H2, H4), 4.01 (dd, *J* = 2.6/9.9, 1H, H6a), 3.85 (dd, *J* = 9.9, 1H, H6b), 3.73 (m, $J_{H3\text{-}H4}$ = 4.4 Hz, 1H, H3), 3.27 (dd, *J* = 12.9 Hz, 1H, H1a), 3.15 (m, 1H, H1b), 2.77 (s, 3H, CH_3). **^{13}C NMR** (400 MHz, D_2O) δ (ppm) 86.0 (C3), 78.7 (C4), 77.5 (C5), 73.5 (C6), 66.7 (C2), 52.1 (C1), 33.5 (CH_3). **HRMS:** m/z calc. for $[M+H]^+$ $C_7H_{16}NO_4^+$: 178.1079; found: 178.1072.

*Spectral data of **9***

*^{1}H NMR spectra of **9***

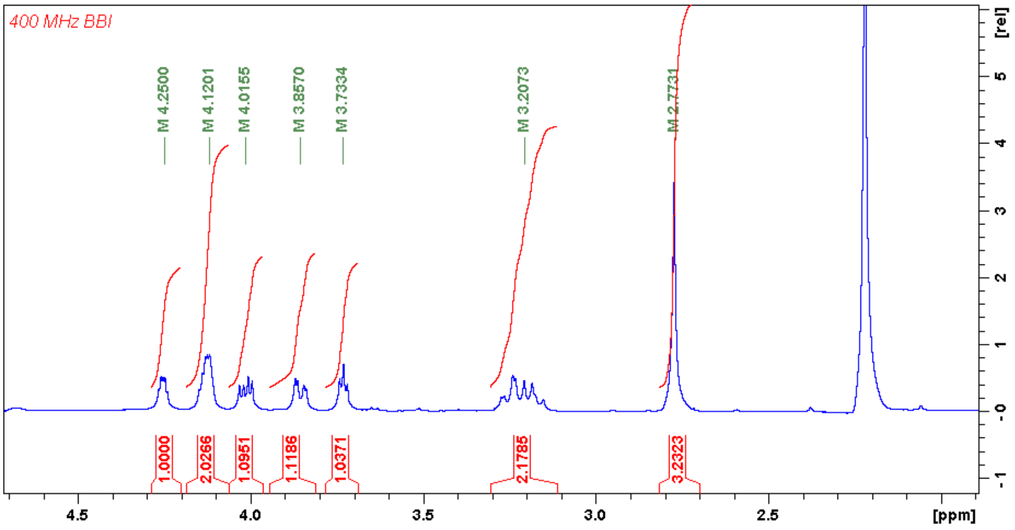

* 400 MHz, D_2O, pH 4.0

*HSQC ^{1}H-^{13}C correlation map of **9***

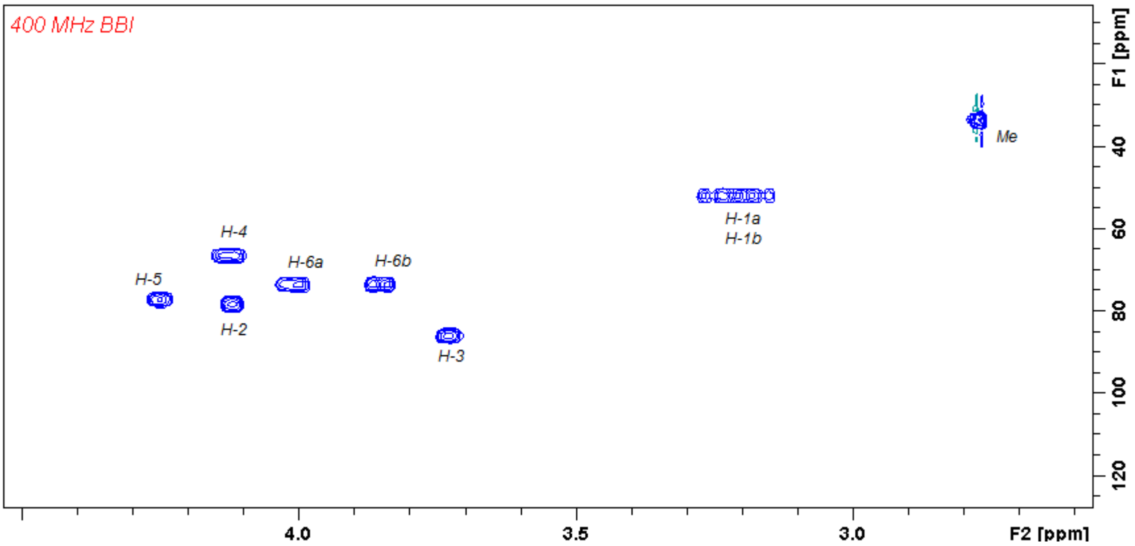

* 400 MHz, D_2O, pH 4.0

3,6-Anhydro-L-galactitol (**11**)[1]

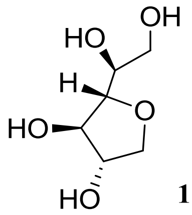

11

Synthesis

Commercial agar (5 g) was first dissolved in hot (≈90 °C) H_2O (450 mL) and then 1 M TFA solution (50 mL) was added in one portion. The resulting mixture was heated at 80 °C for 3 h, cooled to room temperature, diluted with H_2O (400 mL), and then concentrated. The resulting residue was coevaporated with methanol three times to give a syrup. This material was dissolved in H_2O (50 mL), cooled to 0 ºC and then $NaBH_4$ (0.95 g, 25.0 mmol) was added in one portion. The resulting mixture was stirred at room temperature for 1 h, diluted with AcOH (≈4.0 mL, until pH ≈4.0 was reached), stirred for an additional 10 min, and concentrated. The residue was coevaporated with MeOH (3 x 50 mL) to give a yellow syrup. This crude material was dissolved in 2M TFA solution (125 mL) and the resulting mixture was heated at 120 °C for 3 h, cooled to room temperature, diluted with H_2O (100 mL), and then concentrated. The resulting residue was coevaporated with methanol three times to give a syrup. This material was dissolved in H_2O (50 mL), cooled to 0 °C and then $NaBH_4$ (0.95 g, 25.0 mmol) was added in one portion. The resulting mixture was then stirred at room temperature for 1 h, diluted with AcOH (≈4.0 mL, pH ≈ 4.0), stirred for an additional 10 min, and concentrated to give a dark-brown syrup. This material was suspended in MeOH (15 mL) and acetone (50 mL), and then filtered through a glass-sintered filter. The filtrate was concentrated to afford a residue that was diluted with H_2O (50 mL) and treated with Amberlite IRA 410 OH^- form (100 mL). The resulting mixture was stirred for 10 min, filtered through a glass-sintered filter, and washed thoroughly with H_2O (150 mL). The combined filtrates were then treated with Amberlite IR120 (H^+ form 100 mL), stirred for 10 min, filtered through a glass-sintered filter, and washed thoroughly with H_2O (200 mL). The combined filtrates were concentrated to give a dark yellow crude material (1.5 g) mainly constituted of the anhydro alditol.

---

[1] The nomenclature of compound **11** was maintained according to its precursor. However, the correct nomenclature for this compound is 1,4-anhydro-*D*-galactitol

Physical and spectral assignment

^{1}H NMR (400 MHz, D_2O) δ (ppm) 4.22 (m, 1H, H5), 4.11 (m, 1H, H4), 3.99 (dd, *J* = 4.6/10.0 Hz, H6a), 3.82 (m, 2H, H2 and H6b), 3.72 (dd, *J* = 4.9 Hz, 1H, H3), 3.68 (m, 1H, H1a), 3.61 (m, 1H, H1b). **^{13}C NMR** (400 MHz, D_2O) δ (ppm) 85.3 (C-3), 78.9 (C-4), 77.6 (C-5), 73.3 (C-6), 71.9 (C-2), 63.6 (C-1). **HRMS:** m/z calc. for $[M+Na]^+$ $C_6H_{12}O_5Na^+$: 187.0582; found: 187.0580.

*Spectral Data (**11** - crude material)*

*^{1}H NMR spectra of **11***

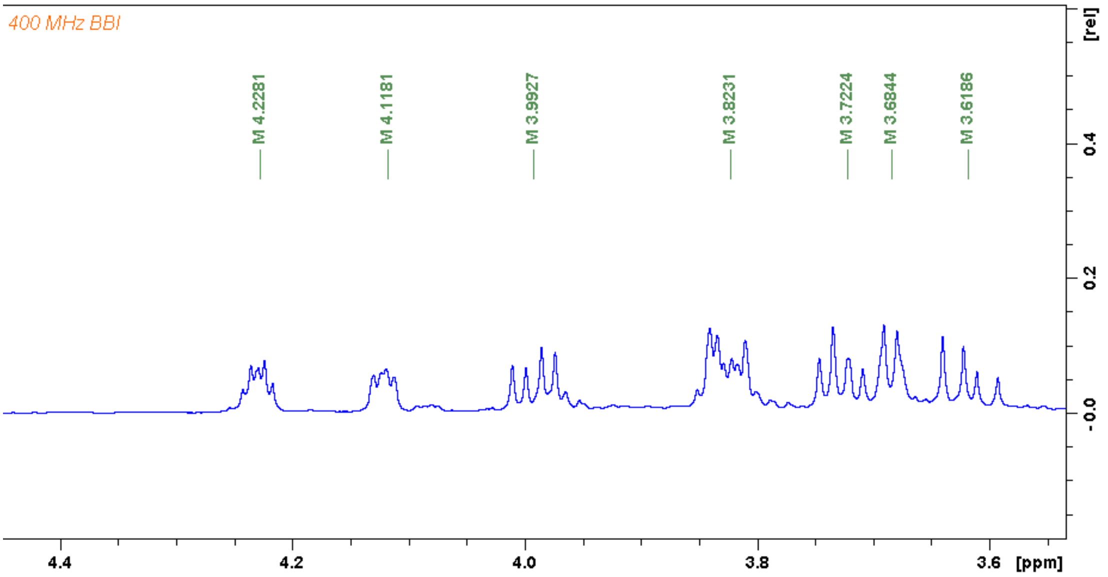

*HSQC ^{1}H-^{13}C correlation map of **11** (crude material)*

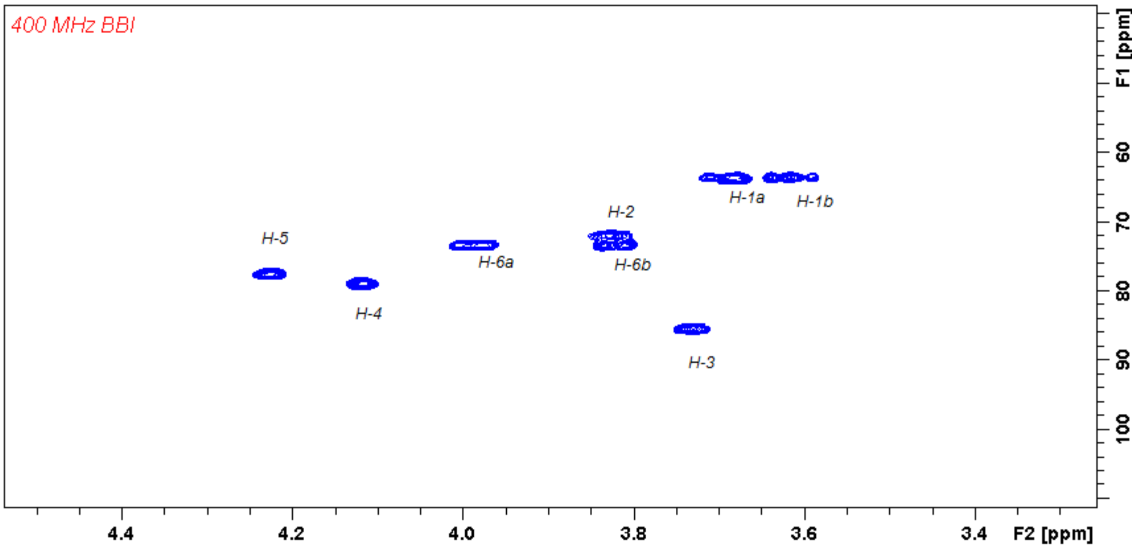

* 400 MHz, D_2O.

2,5-Anhydro-L-lyxitol (**12**)

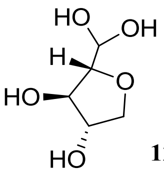

12

Synthesis

The starting material 3,6-anhydro-L-galactitol (**11**, 250 mg, 1.5 mmol) was dissolved in water (3 mL) first, then methanol (3 mL) was added and the mixture was placed in an ice bath. Sodium metaperiodate (330 mg, 1.5 mmol) was added portion wise and the reaction was conducted under vigorous stirring until complete consumption of the starting material was observed by TLC (EtOAc/MeOH/H_2O 3:2:1). The reaction mixture was filtered through a celite pad, washed with methanol and dried under reduced pressure affording crude material containing **12** as major compound.

Physical and spectral assignment

^{1}H NMR (400 MHz, D_2O) δ (ppm) 5.05 (d, 6.2 Hz, 1H, H1), 4.22 (m, 1H, H4), 4.16 (br, 1H, H3), 4.00 (dd, *J* = 4.0/10.1, 1H, H5a), 3.86 (dd, *J* = 0.8/10.1 Hz, 1H, H5b), 3.65 (m, 1H, H2). **^{13}C NMR** (400 MHz, D_2O) δ (ppm) 89.9 (C-1), 87.9 (C-2), 78.2 (C-3), 77.1 (C-4), 73.5 (C-5). HRMS: m/z calc. for $[M+K]^+$ $C^5H^{10}O^5K^+$: 189.0165; found: 189.0246.

*Spectral data (**12** - crude material)*

*^{1}H NMR spectra of **12** (crude material)*

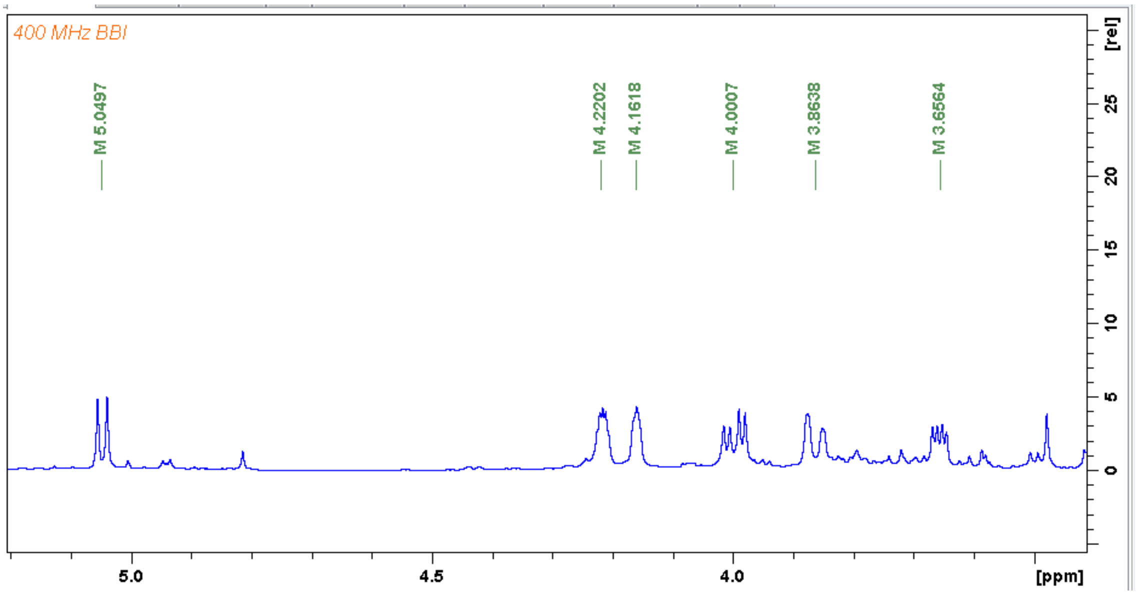

*HSQC ^{1}H-^{13}C correlation map of **12** crude material*

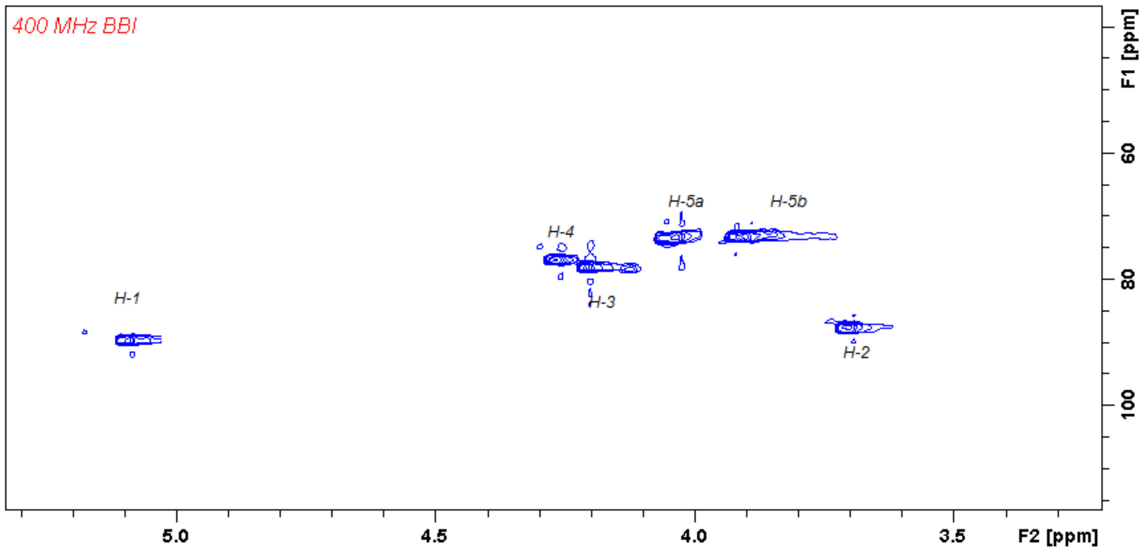

* 400 MHz, D_2O.

1-Deoxy-1-(*N*-methylamino)-2,5-anhydro-L-lyxitol **13**

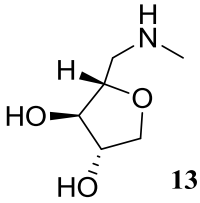

13

Synthesis

Crude material containing aldehyde hydrate **12** (150 mg, 1.0 mmol) was dissolved in water (15 mL) then methylamine hydrochloride (20 equiv, 20 mmol, 1.33 g) was added and homogenized. pH 11 was adjusted with TEA and finally sodium cyanoborohydride (2 equiv, 2.0 mmol, 133 mg) was added in a single portion. The flask was tightly closed immediately. This mixture was placed in a 100 °C glycerin bath and stirred for 5 hours. Reaction workup and purification were done as for the primary aminoglycoside **3** synthesis. After chromatography the compound was obtaine as a white solid after lyophilization (32 mg, 22% molar yield from agarose).

Physical and spectral assignment

$[\alpha]_D$: -12.0 (c 1.0, H_2O). **^{1}H NMR** (400 MHz, D_2O) δ (ppm) 4.18 (dd, J= 1.6/3.6 Hz, 1H, H4), 3.97 (m, 2H, H5a and H3), 3.95 (m, J_{H2-H3} = 3.0 Hz, 1H, H2), 3.84 (dd, *J* = 1.6/10.1 Hz, H5b), 3.08 (m, 2H, H1a and H1b), 2.57 (CH_3). **^{13}C NMR** (400 MHz, D_2O) δ (ppm) 81.7 (C-2), 79.1 (C-3), 76,5 (C-4), 73.2 (C-5), 50.9 (C-1), 33.5 (CH_3). **HRMS:** m/z calc. for $[M+H]^+$ $C_6H_{14}NO_3^+$: 148.0974; found: 148.0920.

Spectral data of ***13***

^{1}H NMR spectra of ***13***

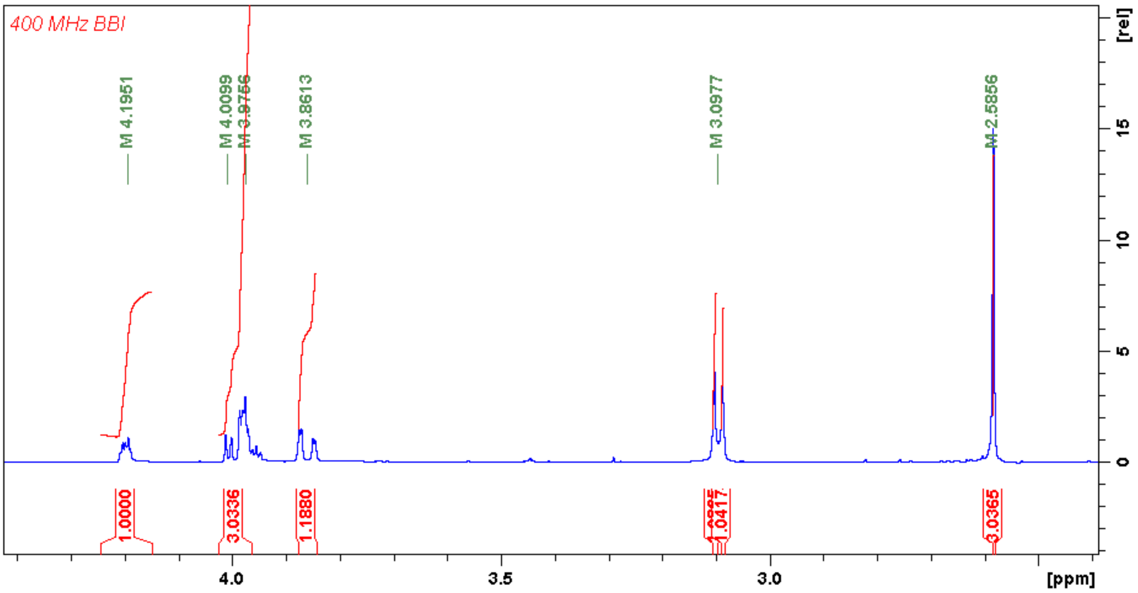

* 400 MHz, D_2O, pH 4.0

HSQC ^{1}H-^{13}C correlation map of ***13***

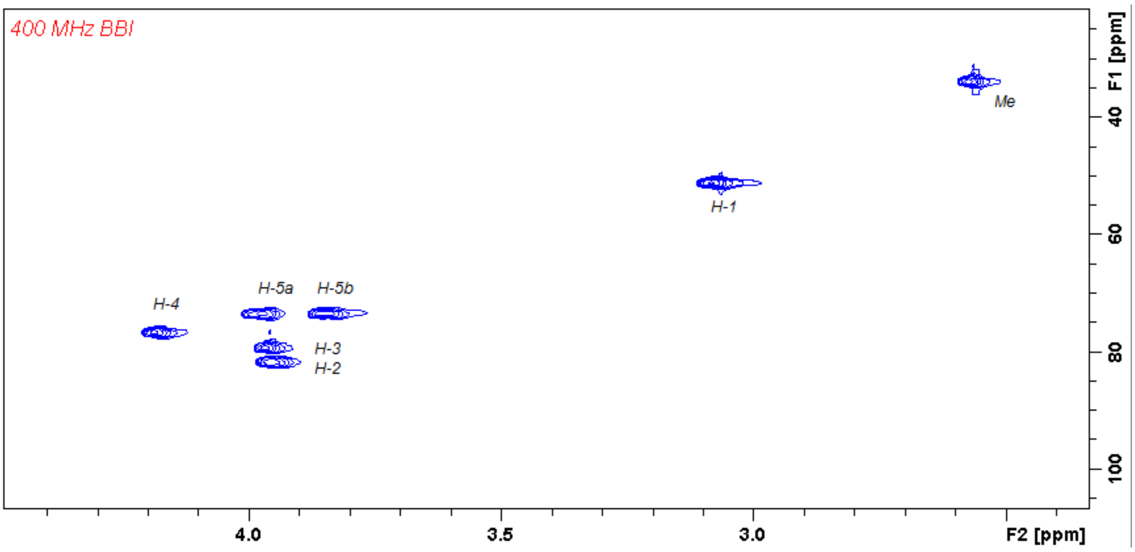

* 400 MHz, D_2O, pH 4.0

Complete table of NMR assignment for compounds **3**,**7** and **8**.

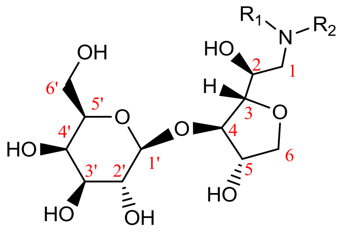

Compound 3: R^1= H R^2 = H

Compound 7: R^1= Me R^2= H

Compound 8: R^1= Me R^2= Me

**Compound**	**3**		**7**		**8**	
**Position**	**^{1}H**	**^{13}C**	**^{1}H**	**^{13}C**	**^{1}H**	**^{13}C**
1a	3.27 (dd, 3.6)	42.9	3.28 (dd, 3.6)	51.9	3.33 (m)nd	59.7
1b	3.12 (dd, 8.8)	42.9	3.22 (dd, 9.0)	51.9	3.33 (m)nd	59.7
2	4.18 (dt,3.6/8.8)	67.2	4.24 (dt, 3.6/9.0)	66.0	4.33 (dt, 3.9/9.2)	64.9
3	3.87 (m)nd	85.0	3.87 (m)nd	84.3	3.84 (dd, 3.9/4.9)	84.1
4	4.32 (ddd, 0.5/2.5/4.9)	85.9	4.32 (ddd, 0.5/2.5/4.9)	85.9	4.31 (ddd, 0.5/2.5/4.9)	85.9
5	4.42 (dt, 2.5/4.7)	76.0	4.42 (dt, 2.5/4.7)	76.0	4.42 (dt, 2.5/4.7)	76.0
6a	4.00 (dd, 4.7/10.2)	73.8	4.00 (dd, 4.7/10.2)	73.8	4.00 (dd, 4.7/10.2)	73.8
6b	3.88 (m)nd	73.8	3.88 (m)nd	73.8	3.88 (dd, 2.5/ 10.2)	73.8
1’	4.57 (d, 7.8)	103.0	4.57 (d, 7.8)	103.0	4.57 (d, 7.8)	103.0
2’	3.51 (dd, 7.8/9.9)	71.4	3.51 (dd, 7.8/9.9)	71.4	3.51 (dd, 7.8/9.9)	71.4
3’	3.66 (dd, 3.4/9.9)	73.3	3.66 (dd, 3.4/9.9)	73.3	3.66 (dd, 3.4/9.9)	73.3
4’	3.92 (dd, 0.7/3.4)	69.4	3.92 (dd, 0.7/3.4)	69.4	3.92 (dd, 0.7/3.4)	69.4
5’	3.71 (m)	76.2	3.71 (m)	76.2	3.71 (m)	76.2
6a’/6b’	3.78 (dd, 5.9)	62.0	3.78 (dd, 5.9)	62.0	3.78 (dd, 5.9)	62.0
CH_3			2.77 (s)	33.7	2.92 (s)	42.0

Data acquired in D_2O, pH 4.0. Given values in δ ppm. Multiplicity and coupling constants (*J*) in Hz are given in brackets. ndCoupling constant not determined due to signal overlapping.
